# Biophysical Analysis of Vip3Aa Toxin Mutants Before and After Activation

**DOI:** 10.3390/ijms252211970

**Published:** 2024-11-07

**Authors:** Pongsatorn Khunrach, Wahyu Surya, Boonhiang Promdonkoy, Jaume Torres, Panadda Boonserm

**Affiliations:** 1 Institute of Molecular Biosciences, Mahidol University, Salaya, Phuttamonthon 73170, Nakhon Pathom, Thailand; frongpongsatorn@gmail.com; 2 School of Biological Sciences, Nanyang Technological University, 60 Nanyang Drive, Singapore 637551, Singapore; wsurya@ntu.edu.sg; 3 National Center for Genetic Engineering and Biotechnology, National Science and Technology Development Agency, 113 Phahonyothin Road, Khlong Luang 12120, Pathum Thani, Thailand; boonhiang@biotec.or.th

**Keywords:** *Bacillus thuringiensis*, Vip3A, mass photometry, sedimentation velocity, differential static light scattering, insecticidal protein

## Abstract

Cry toxins from *Bacillus thuringiensis* are effective biopesticides that kill lepidopteran pests, replacing chemical pesticides that indiscriminately attack both target and non-target organisms. However, resistance in susceptible pests is an emerging problem. *B. thuringiensis* also produces vegetative insecticidal protein (Vip3A), which can kill insect targets in the same group as Cry toxins but using different host receptors, making the combined application of Cry and Vip3A an exciting possibility. Vip3A toxicity requires the formation of a homotetramer. Hence, screening of Vip3A mutants for increased stability requires orthogonal biophysical assays that can test both tetrameric integrity and monomeric robustness. For this purpose, we have used herein for the first time a combination of analytical ultracentrifugation (AUC), mass photometry (MP), differential static light scattering (DSLS) and differential scanning fluorimetry (DSF) to test five mutants at domains I and II. Although all mutants appeared more stable than the wild type (WT) in DSLS, mutants that showed more dissociation into dimers in MP and AUC experiments also showed earlier thermal unfolding by DSF at domains IV–V. All of the mutants were less toxic than the WT, but toxicity was highest for domain II mutations N242C and F229Y. Activation of the protoxin was complete and resulted in a form with a lower sedimentation coefficient. Future high-resolution structural data may lead to a deeper understanding of the increased stability that will help with rational design while retaining native toxicity.

## 1. Introduction

*Bacillus thuringiensis* (Bt) is an aerobic, spore-forming, gram-positive bacterium that is commonly found in soil, insect hosts, plants, and aquatic environments [1]. Bt produces highly potent insecticidal proteins through its various growth phases. The best known are crystal (Cry) and cytolytic (Cyt) toxins, together referred to as δ-endotoxins, synthesized during sporulation. These toxins confer pathogenicity against larvae of diverse insect orders, including Lepidoptera, Coleoptera, Hymenoptera, and Diptera, as well as nematodes [2,3]. In fact, Cry toxins are the most common ingredient in commercial Bt-based biopesticides, employed in agriculture as an alternative to chemical insecticides [4]. However, repeated exposure to Cry proteins leads to resistance [5,6,7,8], prompting the search for alternatives [9].

Some Bt isolates can also produce other toxins that, in contrast to δ-endotoxins, are secreted into the growth medium during vegetative growth, such as the vegetative insecticidal proteins (Vip) [10,11]. Vip proteins are classified into three groups: Vpa, Vpb, and Vip [11]. Within Vip toxins, Vip3 is the largest group. Vip3 members share no structural or sequence similarities with Cry proteins. Despite of this, they are also effective against species of Lepidoptera [10] although targeting different binding sites. Thus, they can potentially complement Cry toxins in resistance management and crop protection. Indeed, a synergistic effect has been observed in laboratory experiments [12,13,14,15]; therefore, Vip3 toxins are regarded as a new generation of insecticidal proteins [9].

Co-administration of Vip3 and Cry toxins is available in Bt-based biopesticides, pyramided Bt crops [16,17,18,19] (although cultivation of Bt crops is still banned in many countries [20]), nanoparticles, and as microencapsules in bacterial cells [21,22].

Vip3 proteins have a molecular mass of about 90 kDa and are released as tetrameric soluble inactive protoxins (~360 kDa) in a solution [23]. To become active, Vip3 protoxins need to be proteolytically digested by trypsin or by insect gut proteases to produce two fragments of about 65 kDa and 20 kDa that remain associated in the solution [23,24,25]. After binding to receptors in the brush border of columnar cells, Vip3 proteins exert insecticidal activity through pore-forming activity and apoptosis induction [13,26,27,28,29,30,31,32].

The structure of Vip3 proteins has been obtained recently [33,34,35]. In particular, single-particle cryo-electron microscopy (SP-cryo-EM) has produced structures of both rounder protoxin and more elongated activated toxin [33,34], which therefore may display different sedimentation coefficients despite having the same molecular weight.

Vip3 is divided based on sequence homology into subfamilies such as Vip3A, Vip3B, and Vip3C. Vip3A is the most studied and widely used in pest control, especially in genetically modified crops like Bt corn and Bt cotton. In turn, Vip3A comprises several subtypes, such as Vip3Aa, Vip3Ab, and Vip3Ac, with up to 95% sequence identity. Among these, Vip3Aa is well studied and is very toxic against pests like fall armyworms and cotton bollworms.

In Vip3Aa [34], the five structural domains (I–V) of protoxin assemble into a pyramid-shaped tetramer as a dimer of dimers, mainly via domains I and II. After activation, the α-helices in domain I transition from being part of a spring-loaded N-terminal apex to an extended four-helix coiled coil which presumably interacts with the target membrane via its N-terminal amphipathic helix, α1 [33]. In this activated form, the four monomers adopt an identical conformation and are arranged around a four-fold symmetry axis [33,34]. In Vip3Aa, the cleavage site for activation is at residue K198 [36], between domains I and II, although other alternative cleavage sites have been suggested [37]. The structure of Vip3Bc1 has similar characteristics [33], and in this case the interaction of the activated toxin with the membrane was directly observed by electron tomography.

The second and third helices of domain I form an antiparallel bundle that adopts a different conformation in the two ‘outer’ and two ‘inner’ monomers. Domain II (residues 200–325) is composed of five α helices and plays a structural role, stabilizing the Vip3 oligomer. Domain III interacts and clamps the most N-terminal segment of the protein (residues 14–23) against the core of the tetramer, whereas domains IV and V likely bind carbohydrates and are not required for oligomer formation [33,34,35], but are necessary for in vivo toxicity [37,38].

Although Vip3 proteins are highly effective at killing lepidopteran pests, commercial development is limited due to their low thermal stability. Since they are secreted in a soluble form, they are easily denatured in the field, retaining activity for only one month at 37 °C [39]. Therefore, in the present study, we have sought to enhance Vip3Aa thermal stability and shelf life by replacing amino acids that may affect intra- and inter-monomeric interactions in domains I and II.

**Figure 1:**
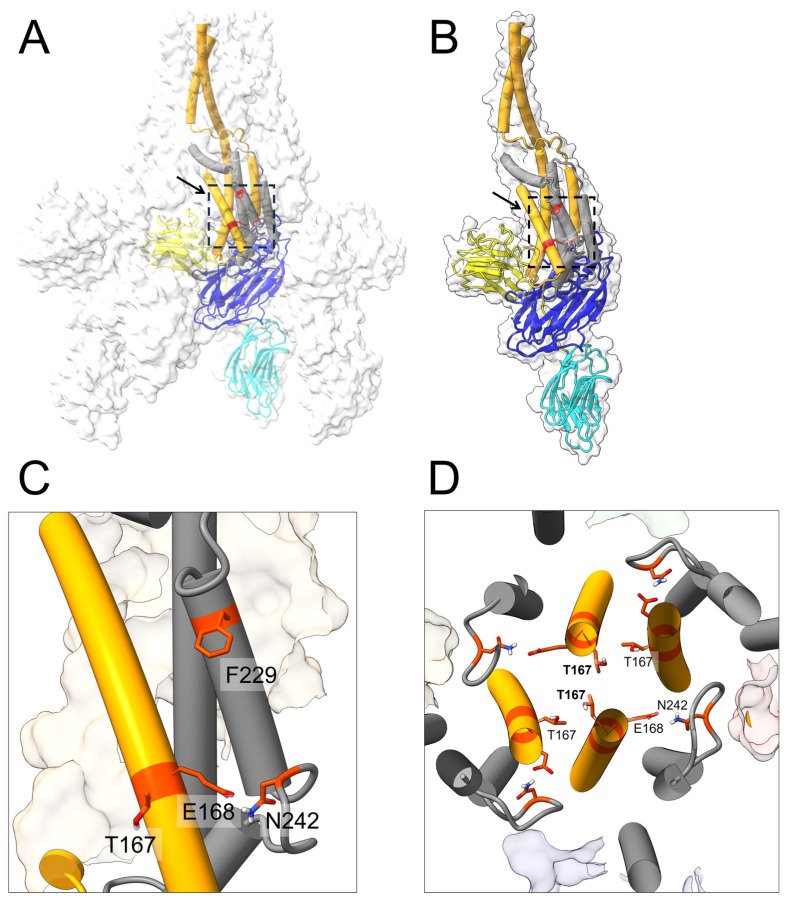
Location of residues mutated in the Vip3Aa protoxin (PDB: 6TFJ). (**A**) Atetrameric model of Vip3Aa, where one of the monomers is shown in color and the other three only as a transparent surface. The four residues mutated are indicated (red orange) and highlighted with a dotted rectangle and arrow. The five domains (I–V) of the toxin monomer are color-coded in orange (1–198), grey (199–325), blue (326–536), yellow (537–675), and cyan (676–789), respectively. (**B**) Same as (**A**) but showing a single monomer. (**C**) A close-up of the four residues mutated, seen from the tetrameric interface, where N242, T167, and E168 were mutated to Cys and F229 to Tyr. (**D**) The top view of the protoxin tetrameric interface, where two T167 residues are in close proximity (bold) whereas the other two are too far away to interact. Nearby residues E168 and N242, which may form interactions, are also shown.

The selection of mutated residues was based on their perceived importance for structural stability, as suggested for Vip3Aa [34]. For example, T167 (domain I) may generate a network of inter-monomeric interactions in the core of both C2-symmetric protoxin (Figure 1D) and C4-symmetric activated toxin [34]. F229 (domain II) interacts with residues in the central part of the domain I α4 helix of the neighboring monomer, forming a hydrophobic pocket that appears to keep α4 attached to the core of the tetramer upon needle formation [34]. Finally, E168 (domain I) and N242 (domain II) may form intra-monomeric interactions to stabilize both domains I and II during protein remodeling. All of these residues, except residue 229, were changed to Cys. In the case of a double mutant formed by E168 and N242, formation of a disulfide bond was expected that would produce a more stable toxin. These mutants were analyzed using a combination of biophysical techniques to determine their stability before performing toxicity assays. These experiments contribute to the rational design of Vip variants to improve efficacy in the field [24,38,40,41,42,43,44,45].

## 2. Results

### 2.1. Mutations

The Vip3Aa toxin gene was mutated at four residues to generate a total of five mutants (one of the mutants contained a double mutation). These four mutations were located in domain I (T167, E168) or domain II (N242, F229) (see Figure 1).

### 2.2. Electrophoresis and Mass Photometry (MP)

**Figure 2:**
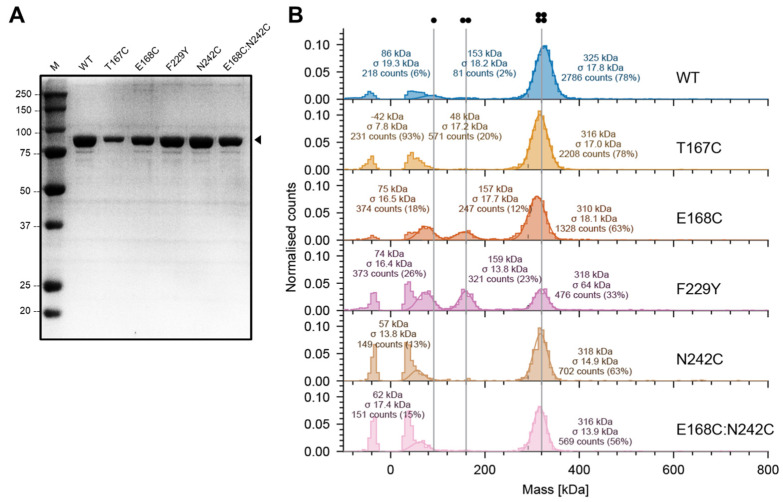
Molecular mass of Vip3Aa protoxin samples measured using mass photometry. (**A**) SDS−PAGE gel corresponding to freshly purified WT Vip3Aa and mutants. The black arrow indicates the band with expected molecular mass for the monomer. (**B**) Mass photometry histograms (particle count) at the indicated mass were fitted to Gaussian distributions (solid lines). Symmetrical peaks centered at 0 kDa are typical noise peaks and were not fitted. Vertical grey lines are shown to guide the eye and indicate the expected mass of monomer, dimer, and tetramer species. For T167C, the peak at 48 kDa could not be fitted into two peaks like for the other mutants. Percentages shown are based on total counts.

After expression and purification, both WT and mutant protoxins produced a single band in SDS gels, with a molecular mass consistent with the expected 86 kDa monomer (Figure 2A). We note that despite the nominal load being the same (2.5 μg), T167C apparently contained less protein. Since the tetrameric form of the protoxin is not captured in SDS gels, the samples were examined using mass photometry (Figure 2B), a new technology that uses light interference to measure single particle mass and which provides direct information on particle heterogeneity in an aqueous solution, i.e., without detergent [46]. Whereas all of the samples showed the expected band consistent with tetramers (~320 kDa), mutants E168C and F229Y also showed a significant proportion of dimers, indicating a higher tendency of the tetramer to dissociate in these mutants. These two samples also contained a significant population with masses lower than expected for monomers, merged with the noise peak (symmetrical peaks centered at 0 kDa), suggesting monomer degradation (the lower limit of detection for MP is ~40 kDa). It is also noticeable that for all mutants, the particle count for the tetramer was lower than for the WT, especially for the domain II mutants F229Y and N242C and the double mutant, with about 25% of the counts relative to the WT. We speculate that this is due to the formation of very large aggregates in these mutants before measurement, which cannot be detected by mass photometry (>5 MDa). Small errors during the necessary dilutions of the sample (2 µL into 18 µL) may also contribute to these variations. In CD spectra, only minor differences were observed in the near UV range (Figure S1).

### 2.3. Tetramer Stability and Activation Observed by AUC-SV

**Figure 3:**
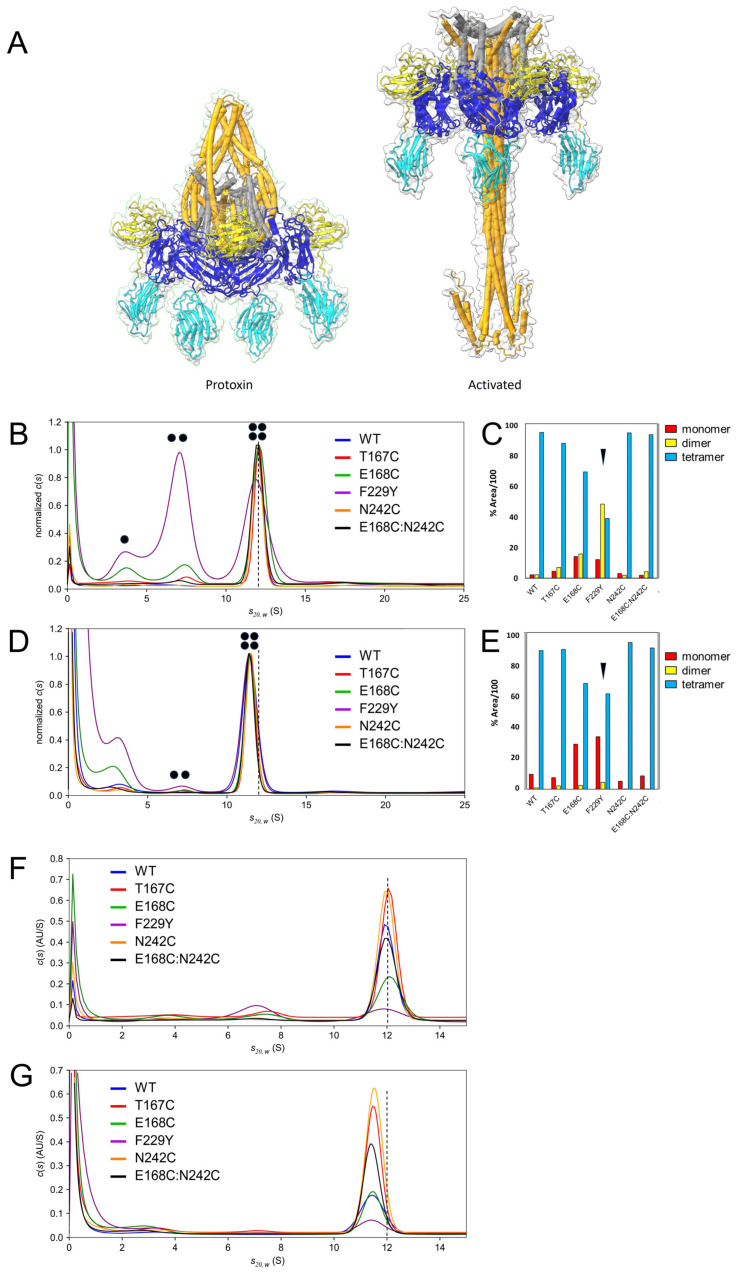
SV analysis of Vip3A protoxin and activated toxin. (**A**) A comparison of the tetrameric shapes of the toxin before (PDB: 6TFJ) and after activation (PDB: 6TFK). In the latter, the N-terminal fragment is missing and only reached residue 95, and therefore it was extended up to residue 25 using the predicted AF2-structure of the domains I–III (overlayed with the experimental structure). (**B**,**C**) A c(s) plot of Vip3A protoxin at 0.5 mg/mL in Tris buffer at 20 °C normalized by band height (**B**) and proportion of different oligomers (**C**) calculated from the relative area under the bands in (**B**). (**D**,**E**) Same as (**B**,**C**), but for the Vip3A activated toxin. (**F**) The c(s) plots of the protoxin, with the y-axis not normalized. (**G**) Same as (**F**) for the activated toxin. The dotted line is shown to guide the eye. Dots in panels (**B**,**D**) represent number of monomers in the oligomer.

We performed an AUC study of the protoxin mutants in sedimentation velocity (SV) mode, which can also provide information on possible unwanted dissociation of tetramers into dimers or monomers (see the raw data in Figures S2 and S3). The same experiment was conducted after protoxin activation with trypsin, where the cleaved parts remain associated and particles retain their original mass but the tetramer converts into a more elongated shape (Figure 3A). Thus, the different hydrodynamic radius should result in a lower sedimentation coefficient.

The normalized c(s) plots of the protoxin (Figure 3B) showed the protoxin tetramer (~12S) and bands assigned to dimers (~7S) and monomers (~3.5S), especially for F229Y and E168C, consistent with the MP data in Figure 2. In particular, mutant F229Y showed an almost equal proportion of monomers distributed into tetramers and dimers (Figure 3C). After activation, a lower sedimentation coefficient (~11.5S) was observed in the WT and mutants (Figure 3D), confirming that all samples were activated. However, the population of dimers in Figure 3B almost disappeared. Instead, a larger band at ~3S and the higher intensity close to 0S suggest the presence of smaller fragments resulting from degradation by trypsin. 

Incidentally, cryo-EM results have suggested that, despite a high efficiency of digestion (>95% according to the PAGE gel), conversion to a needle-like shape after activation is not complete; ~30% of molecules did not change conformation [33,34]. In contrast, our AUC results show that the activated toxin preparation is homogeneous, suggesting a full conversion to a more elongated shape. AUC is the gold standard for the study of mass and shape in a solution since it does not require calibration, labeling or interaction with matrices. Therefore, we hypothesize that this discrepancy may be a result of an artefact created by the interaction of these particles with the grid surface in the electron microscope, although this requires a deeper investigation. 

When the plots in panels B–D were not normalized, this allowed comparison of relative protein abundance in the AUC cell between samples (Figure 3F,G). For the protoxins (Figure 3F), F229Y and E168C showed a lower concentration whereas the most abundant tetramer was that of T167C and N242C. This is despite all samples being initially loaded at the same concentration as measured by absorbance. Similar results were obtained for the activated sample (Figure 3G). This loss of protein may be due either to degradation of the monomers, as shown by the high intensity below 1S in most samples, or to higher aggregation that results in protein accumulation at the bottom of the cell. In summary, the best-behaved mutants in terms of protein loss and (lack of) tetrameric dissociation were T167C and N242C.

### 2.4. Thermal Stability Measured by Differential Static Light Scattering (DSLS)

**Figure 4:**
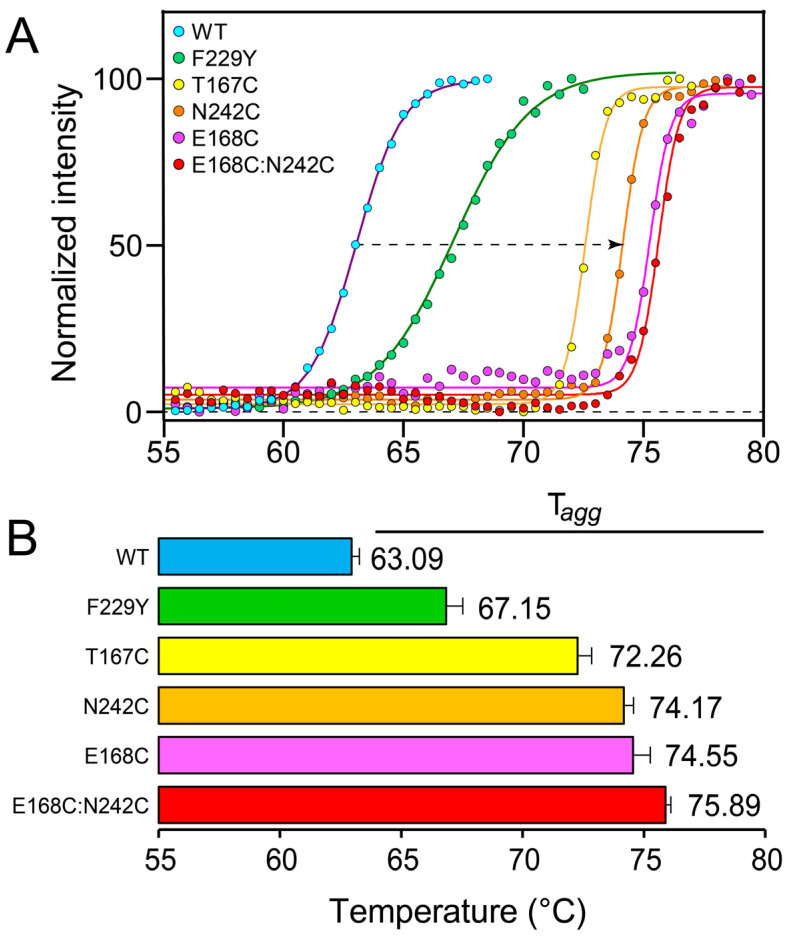
Thermal aggregation curves of protoxin WT Vip3Aa and mutants. (**A**) Light scattering intensity was normalized, plotted as a function of temperature, and fitted to the Boltzmann equation by non-linear regression to obtain the temperature of aggregation, T_agg_. Each curve is a representative of three independent experiments conducted. The arrow indicates the increase in aggregation temperature between the WT and mutant N242C (~11.5 °C). (**B**) Average T_agg_ values, with error bars representing one SD (*n* = 3).

The thermal stability of the protoxin samples was tested using DSLS in a Stargazer-386 machine, which measures the aggregation of the protein with temperature. In all mutants, scattering increase occurred at a higher temperature than in the WT (Figure 4), even for F229Y, which showed a larger dissociation into dimers (Figure 2B and Figure 3B). This suggests that the thermal stability observed here does not correlate with tetrameric integrity. The T_agg_ for other mutants was more than 10 °C higher than the WT, in particular E168C, N242C and the double mutant E168C:N242C. The observed increase in T_agg_ is comparable to those obtained in a mutagenesis study to improve the thermal stability of viral and mammalian proteins [47].

### 2.5. Thermal Stability Measured by Differential Scanning Fluorimetry (DSF)

**Figure 5:**
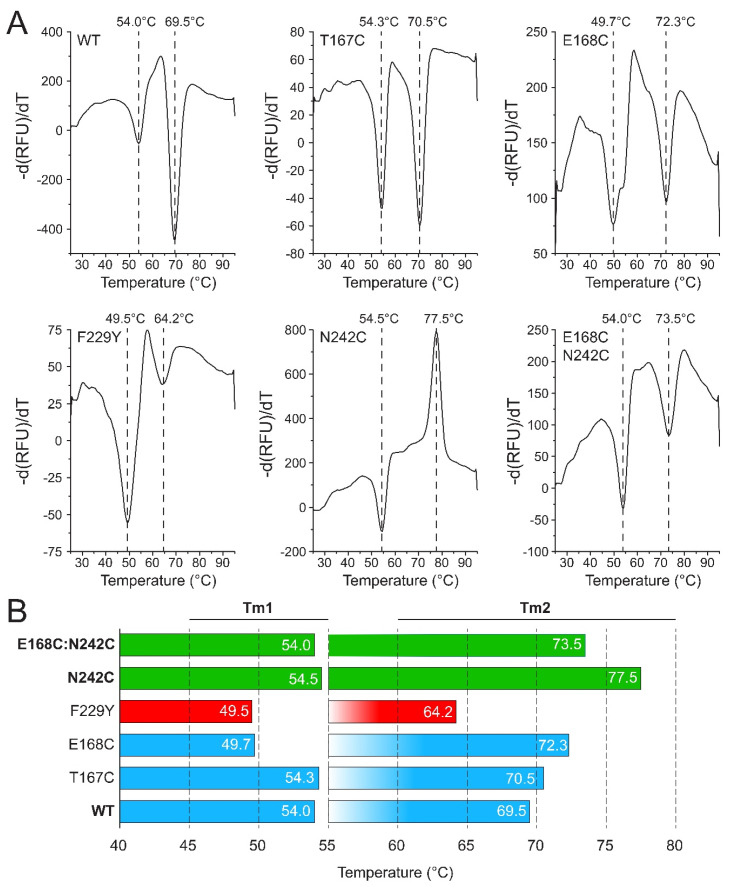
Thermal denaturation shift assay. (**A**) Melting curves of wild-type Vip3Aa and mutants. The melting temperatures (indicated) are marked by vertical dashed lines. Tm1 and Tm2 values are summarized in (**B**) with colors to guide the eye: green: more stable than the WT; blue: similar to the WT; red: both Tm1 and Tm2 lower than in the WT.

The thermal stability of the protoxin samples was also measured by DSF using SYPRO Orange dye, which binds the protein hydrophobic regions that become exposed during thermal unfolding (Figure 5). The samples showed the two melting temperatures described previously, which correspond to the unfolding of domains IV–V (lower temperature Tm1) and domains I–III (higher temperature Tm2) [48]. Tm1 for the mutants was close to that of the WT, except in E168C and F229Y, where it was lower by ~4 °C. These two mutants also showed the largest concentration of dimers in the MP and AUC experiments (Figure 2B and Figure 3B), which suggests that tetramerization stabilizes domains IV–V. For F229Y, Tm2 was also lower than the WT by 5 °C; therefore, it is the least thermostable mutant. For all the other mutants, Tm2 was higher than the WT, in a trend that somewhat correlates with the T_agg_ data (Figure 4B), so that with the exception of the WT, increased stability in DSLS appears to correlate with the stability of domains I–III (Tm2). These results underscore the need to perform multiple and complementary assays to characterize these samples. Looking at Tm2, N242C and the double mutant E168C:N242C were the most stable by DSF (4–8 °C increase vs the WT), consistent with T_agg_ data (Figure 4). We note that the second transition temperature for N242C is marked by a positive peak instead of a negative one. We are at present unable to explain this feature which was reproducible in three different samples.

### 2.6. Toxicity Assay

**Figure 6:**
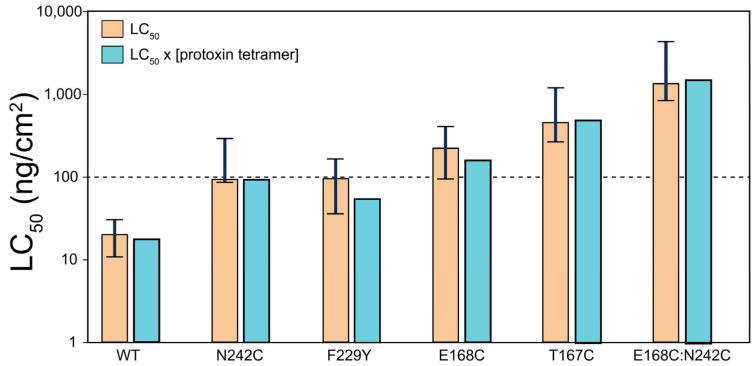
Toxicity against *S. exigua* results. Orange bars: graphical representation in a logarithmic scale of LC_50_, with fiducial limits represented by a vertical bar; cyan bars: LC_50_ normalized for the proportion of protoxin tetramer according to AUC and mass photometry.

The toxicity of the Vip3Aa mutants against *S. exigua* was compared to the WT (Table 1). For clarity, we also represent these results graphically, and in logarithmic scale (Figure 6). It is clear that the WT is the most toxic species, followed by mutants N242C and F229Y (Figure 6, orange). However, since the different samples show varying proportions of tetramer in AUC and mass photometry (see Figure 2B and Figure 3B), we attempted to normalize these results to account for the actual proportion of tetramer protoxin that may be present in the toxicity assay. This was carried out by multiplying the LC_50_ with the estimated fraction of tetramer, i.e., from 1 if all of the sample is tetrameric to 0 if the sample is not tetrameric, obtained from AUC and mass photometry. For example, the mutant F229Y has slightly lower toxicity than N242C (Figure 6, orange), but the amount of tetramer responsible for this toxicity is likely lower than that of N242C, which translates into a lower effective LC_50_ for F229Y than the one observed. Taking this into account, we conclude that the most toxic mutant after the WT was F229Y, i.e., the least stable of the mutants (Figure 4 and Figure 5) and the one that showed more tetramer dissociation (Figure 3). This was followed by N242C, which was also the most stable mutant as found via DSF (Figure 5). The double mutant showed the lowest toxicity despite being one of the most stable mutants (Figure 4 and Figure 5), suggesting that higher stability was obtained at the cost of impaired functionality.

## 3. Discussion

### 3.1. General Objective

The Vip3 family of Bt insecticidal proteins has emerged as an attractive complement to enhance the effect of Cry toxins, by acting synergistically against a wide range of lepidopteran pests for crop protection [10,12,13,14,15]. Commercial development of Vip3 proteins has been successfully demonstrated in transgenic or Bt crops by pyramiding the *vip* and *cry* genes [19], but the low thermal stability of Vip3 proteins restricts their application as formulated biopesticides [39].

### 3.2. Justification of the Residues Chosen

The residues mutated herein are located in one of the two critical clusters (the N-terminal one) identified in Vip3Af by alanine-scan mutagenesis [38], which includes the end of domain I and the whole of domain II, i.e., residues 167–272. In particular, Cys mutation at T167, located at the interface between the four monomers [34], was expected to form inter-molecular disulfide bonds, creating a more stable tetramer than in the wild type. In the protoxin, the experimental structure suggests that T167 forms inter-monomer interactions only with the two ‘internal’ monomers (Figure 1D), whereas in the activated toxin, these interactions may involve the four monomers, with the side chains pointing toward the central axis. The side chains of residues E168 (domain I) and N242 (domain II) of the same monomer are at a close distance, at least in the protoxin structure [34], and a hydrogen bond may contribute to the stabilization of the protoxin, or during the needle formation following proteolytic activation. The introduction of a double Cys mutant at these residues (mutant E168C:N242C) was expected to provide an even stronger covalent interaction. Single Cys mutations (E168C or N242C) were also tested as a control, although a single substitution to cysteine can also establish stronger interactions with nearby amino acids (e.g., by hydrogen bonding, hydrophobic interactions, or aromatic and aliphatic π interactions) yet result in a functional protein. Finally, F229 is located at domain II and interacts with the neighboring monomer, forming a hydrophobic pocket that stabilizes the core of the tetramer upon needle formation [34]. Besides hydrophobic interactions, additional hydrogen bonding was anticipated with mutation F229Y, which could provide stronger intra-monomeric contacts in the tetramer.

### 3.3. Comparison with Experimental Structures

**Figure 7:**
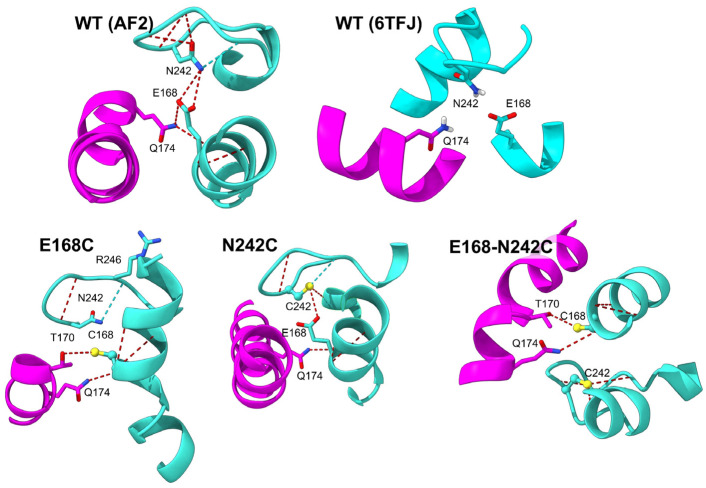
Comparison of the possible interactions of protoxin residues 242 and 168. In the WT AF2-predicted structure, N242 forms hydrogen bonds with E168 of the same monomer (cyan), and the latter forms another via main chain atoms with Q174 of a different monomer (magenta). In the experimental structure (6TFJ), these three residues are close, although too far to form hydrogen bonds. In the E168C mutant, the AF2-predicted model shows that the introduced cysteine can form hydrogen bonds with T170 of a neighboring monomer in addition to the aforementioned interaction with Q174, also present in the AF2-predicted models of mutants N242C and the double mutant. N242C may form three possible interactions with the same monomer, one of which is with E168.

In the protoxin structure of Vip3A [34] (PDB: 6TFJ) there is a possible hydrogen bond between N242 H and E168 O (separated by only 1.8 Å), although in the activated toxin (PDB: 6TFK), this distance increases to 3.2 Å, and N242 only forms a hydrogen bond with R246. In the protoxin structure of Vip3Bc1 [33] (PDB: 6YRF), the distance between N252 (equivalent to N242 in Vip3Aa) and E178 (equivalent to E168 in Vip3A) is too long (3.5 Å) to form H bonds, although N252 forms a hydrogen bond with F254. To ascertain the effects of the mutations introduced in the protoxin or activated structure, we used Alphafold 2 (AF2). However, the AF2-predicted protoxin tetramer has a four-fold symmetry, different from the two-fold symmetry described experimentally [33,34]. This may represent an artefact or an intermediate resulting from activation, since the activated toxin has indeed a four-fold symmetry [33,34]. Nevertheless, in the AF2-based protoxin, N242 appears to be very important for maintaining stability, as it forms hydrogen bonds with both E168 and R246 of the same monomer (Figure 7). 

### 3.4. Thermostability

Vip3A has been reported to unfold via two transitions: domains IV and V unfold at a lower temperature (Tm1) than domains I–III (Tm2) [48]. As expected, mutating key interfacial residues in domains I and II to Cys resulted in increased Tm2. Despite the location of the mutations, Tm1 was reduced in mutants E168C and F229Y, where more tetramer dissociation occurred according to MP and AUC, suggesting a propagation of a mild conformational change between these domains when the tetramer is less stable. The most thermostable mutant (E168C:N242C) according to DSLS was also the second most stable in DSF (after N242C) but was the least toxic. This suggests that the enhanced stability in this double mutant is due to the formation of a disulfide bond between the two residues, which restricts conformational movement and toxicity. In the case of the single mutants, increased stability may be due to the formation of additional interactions with neighboring residues. This can only be ascertained from high-resolution structural data of these mutants which is out of the scope of the present paper. Changes are expected to be structurally very minor, since no differences were observed in the CD far UV spectra, and only minor differences were observed in the near UV spectra, particularly for mutant F229Y.

### 3.5. Rationale for Increased Stability

Both Vip3 protoxin and the trypsin-activated toxin form a stable tetramer that is essential for toxicity [33,34]. Domain I is critical for Vip3 tetramer stability [38,48,49], also contributed to by domains II and III [33,34]. Moreover, a tetrameric Vip3_BA chimera was more resistant to degradation by midgut proteases than the separate monomers, supporting the idea that tetramerization prevents degradation [24]. This is consistent with the enhanced degradation products (and lower abundance of the tetramer) observed for mutants F229Y and E168C, which were also the ones with the lowest Tm2. 

### 3.6. Larvicidal Activity

Larvicidal activity against *S. exigua* was the lowest for the E168C:N242C double mutant, whereas mutant F229Y was one of the most toxic (with N242C). The stability of T167A was clearly higher than the WT in both assays, and its toxicity was one of the lowest. Finally, E168A was more stable in DSLS than the WT but Tm1 was clearly lower. Overall, N242C and F229Y were the mutants that retained more toxicity, and N242C was the most stable one according to DSF. In future work, the toxicity of these mutants could perhaps be increased by inserting mutation M34L in helix α1 of domain I [37]. Indeed, when this mutation was tested against *S. exigua* larvae, LC_50_ was only 4.5 ng/cm^2^ compared to the wild-type toxin (LC_50_ = 19.5 ng/cm^2^), and a similar result was obtained in Vip3Af [45]. Thus, this study is a template for the future investigation of thermally stable and toxic mutants that may be obtained by site directed mutagenesis.

## 4. Materials and Methods

### 4.1. Bacterial Strains, Plasmids, and Media

*Escherichia coli* JM109 carrying the recombinant plasmid pET28b-Vip3Aa64 (Vip3Aa64 accession number: KY883694, hereafter referred to as ‘Vip3Aa’) was obtained from the Biocontrol Technology Research Laboratory, National Center for Genetic Engineering and Biotechnology, Khlong Luang, Thailand. The *vip3Aa* gene was located at the multiple cloning site between *Nde*I, 6xHistidine tag, and prothrombin site at 5′ end and *Xho*I at the 3′ end. *E. coli* DH5α was used as a cloning host. *E. coli* BL21(DE3)pLysS was used as expression host. Luria–Bertani (LB) broth was used as a medium for propagating the recombinant plasmid in *E. coli* DH5α and for Vip3Aa protein production in *E. coli* BL21(DE3)pLysS.

### 4.2. Mutagenesis

The recombinant plasmid pET28b-Vip3Aa64 was used to engineer mutations T167C, E168C, F229Y, N242C, and E168C:N242C using site-directed mutagenesis (primers shown in Table 2). PCR products were treated with *Dpn*I prior to heat shock transformation into *E. coli* DH5α. The extracted plasmids from each mutant clone were verified by Sanger sequencing (1st Base, Singapore). Thereafter, plasmids with the desired mutations were transformed into *E. coli* BL21(DE3)pLysS.

### 4.3. Protein Production and Purification

*E. coli* BL21(DE3)pLysS cells harboring wild-type (WT) or mutant Vip3Aa were grown in LB broth supplemented with 34 μg/mL chloramphenicol and 50 μg/mL kanamycin at 37 °C and stirred at 220 rpm. When OD600 reached 0.4 to 0.6, IPTG (0.4 mM) was added to induce expression. The culture was grown at 25 °C and 220 rpm for another 18–20 h. Cells were harvested by centrifugation (8000× *g* at 4 °C for 5 min). Cell pellets were resuspended in Tris buffer (50 mM Tris-HCl, pH 8.0, 200 mM NaCl) and lysed by ultrasonication with 50% amplitude, with 5 s on/5 s off pulses for 5 min. The soluble fraction was obtained from the supernatant after centrifugation at 12,000× *g* for 10 min and filtration through a 0.45 µm filter. The supernatant containing (His)_6_-tagged Vip3Aa protein was loaded into a Ni–NTA affinity column HiTrap™ Chelating HP (GE Healthcare, Uppsala, Sweden) and eluted with Tris buffer containing 100–250 mM imidazole. Protein fractions were concentrated by ultrafiltration at 4 °C using a Centriprep column (30-kDa cut-off, Amicon, Cork, Ireland), and excess imidazole was removed with a desalting column. The protein collected at every step was analyzed by 12% sodium dodecyl sulfate–polyacrylamide gel electrophoresis (SDS-PAGE).

### 4.4. Proteolytic Activation by Trypsin Digestion

The purified Vip3A protoxin (5 μg) was mixed with trypsin (l-1-tosylamide-2-phenylethyl-chloromethyl-ketone-treated, Sigma, St. Louis, MO, USA) at 5% *w*/*v*, and incubated at 37 °C for 1 h with shaking at 350 rpm. Trypsin digestion was interrupted by freezing at −20 °C the night before its use in the AUC-SV experiment. The activated toxin (10 µL) in wild type or mutants was analyzed by SDS-PAGE, and bands were compared to those of the respective protoxin.

### 4.5. Gel Electrophoresis

Protein samples (2.5 µg) were mixed with 5× SDS-PAGE loading buffer containing 10% SDS, 50% glycerol, 250 mM Tris-HCl pH 6.8, 0.5% bromophenol blue dye, and 500 mM dithiothreitol (DTT). Samples were heated at 95 °C for 10 min before centrifugation (12,000 rpm for 5 min) and loaded into 12% 1.0 mm thick SDS-PAGE gels, together with molecular weight markers (Bio-Rad Precision Plus Protein™ Standards, Hercules, CA, USA). The gels were run at 80 V until the dye front reached the edge of the separating gel. Then, the voltage was increased to 120 V and continued running for approximately 60 min, using TGS (25 mM Tris, 192 mM glycine, 0.1% SDS, pH 8.3) as a running buffer. The gels were stained with Coomassie blue (Bio-Rad, Hercules, CA, USA) and destained using 30% methanol and 10% acetic acid to visualize protein bands. Gel images were captured using Syngene^®^ G:Box gel documentation system, Cambridge, UK.

### 4.6. Analytical Ultracentrifugation Sedimentation Velocity (AUC-SV)

Experiments were performed using a Beckman Coulter XL-I analytical ultracentrifuge (IN, USA) with an An-50 Ti rotor. Protoxin Vip3Aa or trypsin-digested Vip3Aa in 50 mM Tris-HCl at pH 8.0 was diluted to give an absorbance at 280 nm of 0.5. Concentration was measured using a NanoDrop™ OneC spectrophotometer (Thermo Scientific, Waltham, MA, USA) from the absorbance at 280 nm and extinction coefficient calculated using https://web.expasy.org/protparam/ (accessed on 6 June 2023). The samples were loaded into standard double-sector Epon charcoal-filled centerpieces, and the buffer alone was used as a reference. The sample was centrifuged at 35,000 rpm at 20 °C and sedimentation profile was collected every 5–10 min. SV data were analyzed using SEDFIT v. 16.1c software via a continuous c(s) distribution analysis [50]. The data were fitted by minimizing the root-mean-square deviation (rmsd) of the residuals. Sedimentation coefficients were standardized to s_20,w_ using the buffer density (1.00649 g/cm^3^) and viscosity (1.0206 cP) calculated using SEDNTERP v.303 [51]. Partial-specific volume (ῡ) of activated Vip3Aa (0.7293 cm^3^/g) was calculated from the amino acid sequence by using SEDNTERP [51].

### 4.7. Mass Photometry

The molecular mass of protoxin samples (10–20 nM on a tetramer basis) in 50 mM HEPES pH 7.4, 150 mM NaCl, 1 mM EDTA were measured using Refeyn TwoMP (Refeyn, Oxford, UK) following the manufacturer’s instructions. Mass calibration was conducted using BSA (66 kDa, 132 kDa) and Thyroglobulin (660 kDa). Mass histograms were fitted to Gaussian distribution using DiscoverMP v. 2024R1 software provided by the manufacturer (Refeyn, Oxford, UK).

### 4.8. Thermal Aggregation Shift Assay (Differential Static Light Scattering)

Temperature-dependent protein aggregation was measured with differential static light scattering (Stargazer-384, Harbinger Biotech, Markham, ON, Canada) as previously described [52,53] with modifications. Briefly, purified toxin in Tris buffer (50 mM Tris-HCl pH 8.0 and 200 mM NaCl) at 0.5 mg/mL was incubated at room temperature for 15 min, and aliquoted (50 µL) in a clear-bottom 384-well plate (Nunc, Roskilde, Denmark) in triplicate. Mineral oil (50 µL) covered the sample to prevent evaporation. The sample was heated from 25 to 85 °C (0.5 °C/min). Data collected were analyzed using Bioactive software v. 2.1.10 (Harbinger Biotech). Intensities were plotted as a function of temperature and fitted to the Boltzmann equation by non-linear regression. The point of inflection of the fitted curve defined the temperature of aggregation (T_agg_).

### 4.9. Thermal Unfolding Shift Assay (Differential Scanning Fluorimetry)

Temperature-dependent protein unfolding was measured via binding of SYPRO Orange dye (Sigma-Aldrich, St. Louis, MO, USA) on CFX96 Touch Real-Time PCR System (Bio-Rad, Singapore), according to Bio-Rad’s Protein TSA protocol. Briefly, purified protoxins in Tris buffer (50 mM Tris-HCl pH 8.0 and 200 mM NaCl) at 0.3 mg/mL were mixed with 5X SYPRO Orange and aliquoted (20 µL) into a clear 96-well PCR plate (Bio-Rad, Singapore). Lysozyme (10 μM) was used as an internal control, and buffer-only sample as baseline. Each reaction mixture was 20 μL and each sample was prepared in triplicate. The melting curve data were recorded from fluorescence reading every 0.5 °C during a thermal ramp from 25 °C to 95 °C at 3 °C/min. The data were processed and analyzed using CFX Manager v. 3.1 software (Bio-Rad, Singapore). The transition temperatures were determined from the first derivative of the melting curve.

### 4.10. Circular Dichroism (CD)

CD spectra of the protoxin samples (absorbance at 280 nm of 0.5) were collected using Chirascan CD spectrophotometer (Applied Photophysics, Leatherhead, UK) in 10 mm quartz cuvette from 260–360 nm with 1 nm step and 1 s dwell time per point at 25 °C. To enhance the differences between the spectra, data were collected as a function of time for samples at 55 °C.

### 4.11. Insecticidal Activity Against Spodoptera exigua Larvae

The artificial diet used to test insecticidal activity consisted of a mixture of mung bean powder, yeast extract, vitamins, agar, and water [39]. The assay was performed on 1.9 cm^2^ of a 24-well plate by pouring 1 mL of diet and overlaying 50 µL of toxin to produce final concentrations of 30 to 2000 ng/cm^2^. Negative controls used only buffer with no toxin. Two second-instar *S. exigua* larvae were placed into each well, using 16 larvae for each toxin concentration. Larvae mortality was measured after seven-day incubation at room temperature. The LC_50_ values (median lethal concentration) were calculated from three independent experiments by using Probit analysis. The animal study protocol was approved by the Institute of Molecular Biosciences Animal Care and Use Committee (IMB-ACUC) (COA. NO. IMB-ACUC 2021/019).

### 4.12. Alphafold-2 (AF2) Structure Prediction

The protoxin structure (domains I–V) was predicted using a local installation of AF2 (commit 7c9114c, 10 August 2023) [54] using the multimer mode [55] with full dataset and 5 seeds, selecting the top-ranked model. The structure of the ‘activated toxin’ (needle-like) was obtained with AF2 by using just domains I–III of Vip3Aa in ColabFold (AlphaFold2 using Mmseqs2) [56] that uses AlphaFold-2 [54]. The best structure was minimized with Amber (*use_amber* = True). Other parameters were *template_mode* = None, *msa_mode* = MMSeqs2 (Uniref + Environmental), *pair_mode* = unpaired + paired, *model_type* = auto, *num_recycles* = 6, and number of seeds = 3. For each prediction, the best models (rank 1) were selected according to average pLDDT, and complexes were sorted by pTMscore, which reports on the accuracy of prediction within each protein chain. The validity of the models was determined by their low PAE score. Graphical representation was performed in Chimera X [57,58].

## Figures and Tables

**Table 1:** Insecticidal activity. The toxicity of wild-type (WT)Vip3Aa64 and mutants against *S. exigua* larvae determined with concentrations from 0.0625 to 2.0 μg/cm^2^. Mortality was monitored for seven days before LC_50_ was determined. Mutants F229Y and N242C showed the highest toxicity, together with the WT (all in bold).

Vip3Aa64	LC_50_ (ng/cm^2^)	95% Fiducial Limit (ng/cm^2^)	
		Lower	Upper
**WT**	20	11	31
T167C	454	266	1187
E168C	222	94	405
F229Y	95	38	172
N242C	93	90	302
E168C:N242C	1342	782	4164

**Table 2:** Forward (For) and reverse (Rev) primers used for mutagenesis of the *vip3Aa64* gene (mutated residues represented by underlined bases) and their melting temperatures.

Primer’s Name	Sequence (5′-3′)	Tm (°C)
T167C-For	TAACTCTACACTTTGTGAAATTACACCTGC	50
T167C-Rev	GGTGTAATTTCACAAAGTGTAGAGTTAATA	46
E168C-For	TCTACACTTACTTGCATTACACCTGCGTAT	49
E168C-Rev	GCAGGTGTAATGCAAGTAAGTGTAGAGTTA	47
F229Y-For	ATGGTTTTGAATATTACCTTAATACATTCC	52
F229Y-Rev	TGTATTAAGGTAATATTCAAAACCATCCAC	52
N242C-For	ATGGTAGGAAATTGTTTATTCGGGCGTTCA	53
N242C-Rev	CGCCCGAATAAACAATTTCCTACCATTACA	53

## Data Availability

The data presented in this study are available on request from the corresponding author.

## Supplementary Materials

The following supporting information can be downloaded at: https://www.mdpi.com/article/10.3390/ijms252211970/s1.
