# Supplementary material for: Biophysical Analysis of Vip3Aa Toxin Mutants Before and After Activation
Source: Int J Mol Sci. 2024 Nov 7;25(22):11970. doi: 10.3390/ijms252211970 (PMC11594144; doi:10.3390/ijms252211970)
Supplement: Supplementary file 1 [file ijms-25-11970-s001.zip › ijms-3267195-supplementary.pdf]

Supplementary file. Vip3Aa toxin mutants with high thermal stability

Pongsatorn Khunrach et al.

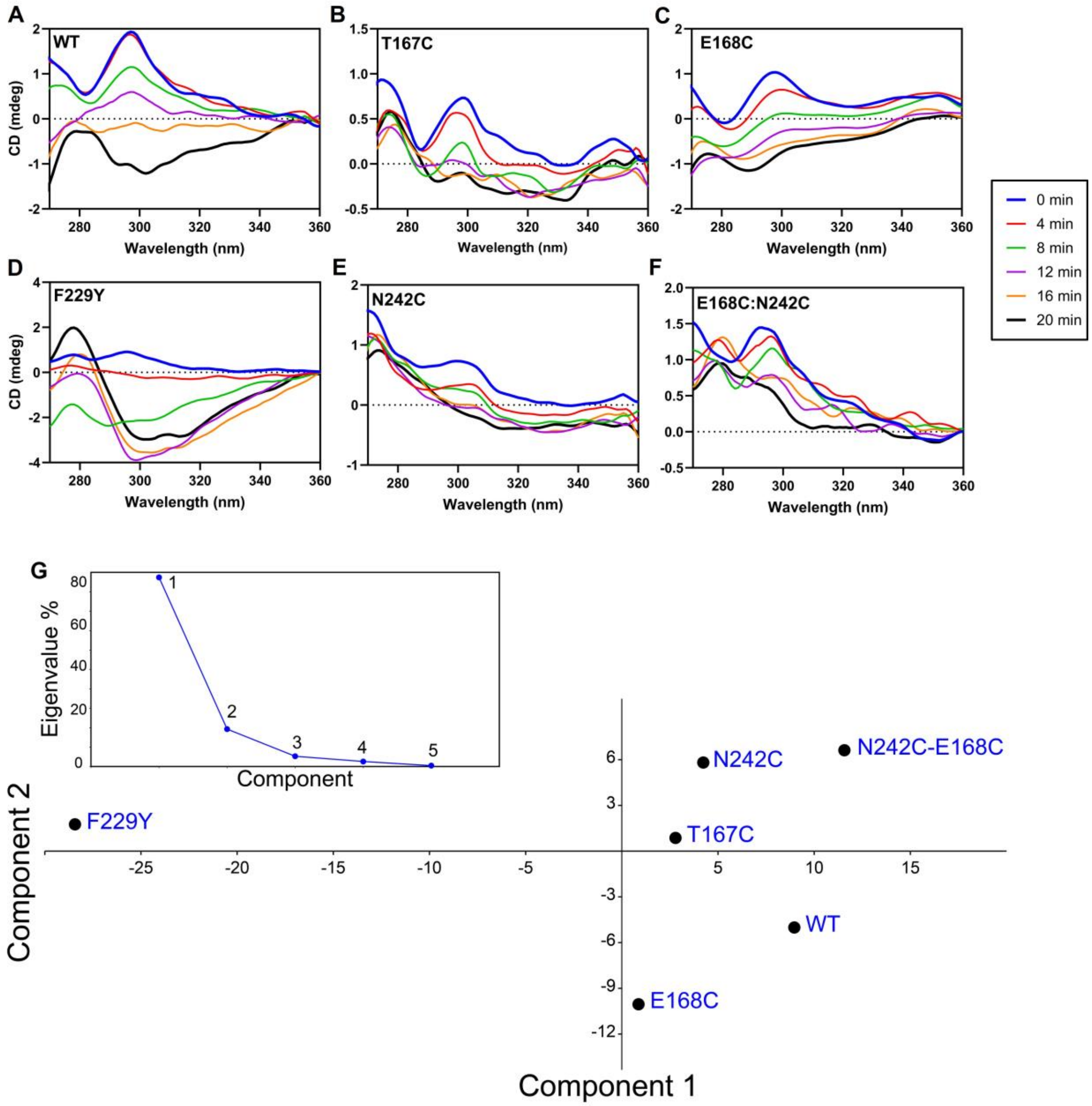

Figure S1. Near UV CD spectra. Data collected all WT and mutants incubated at 55 °C, collected at the minutes shown (A-F); (G) because of the difficult interpretation of these changes, the second derivatives of these spectra were analysed using principal component analysis (PCA).

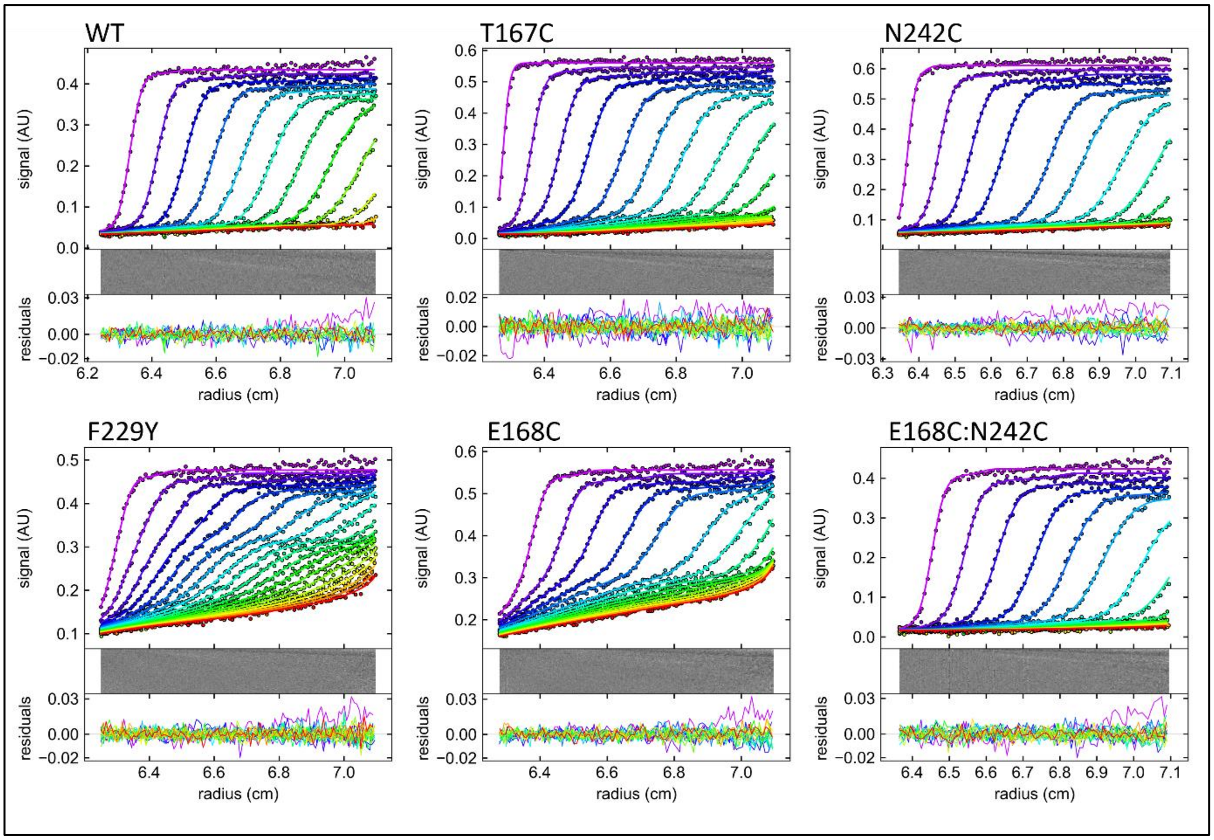

Figure S2. Boundary migration for Vip3Aa protoxin. Raw representative sedimentation velocity data corresponding to the samples indicated. Each boundary is fitted to a c(s) model, and the residuals are shown in the lower panels.

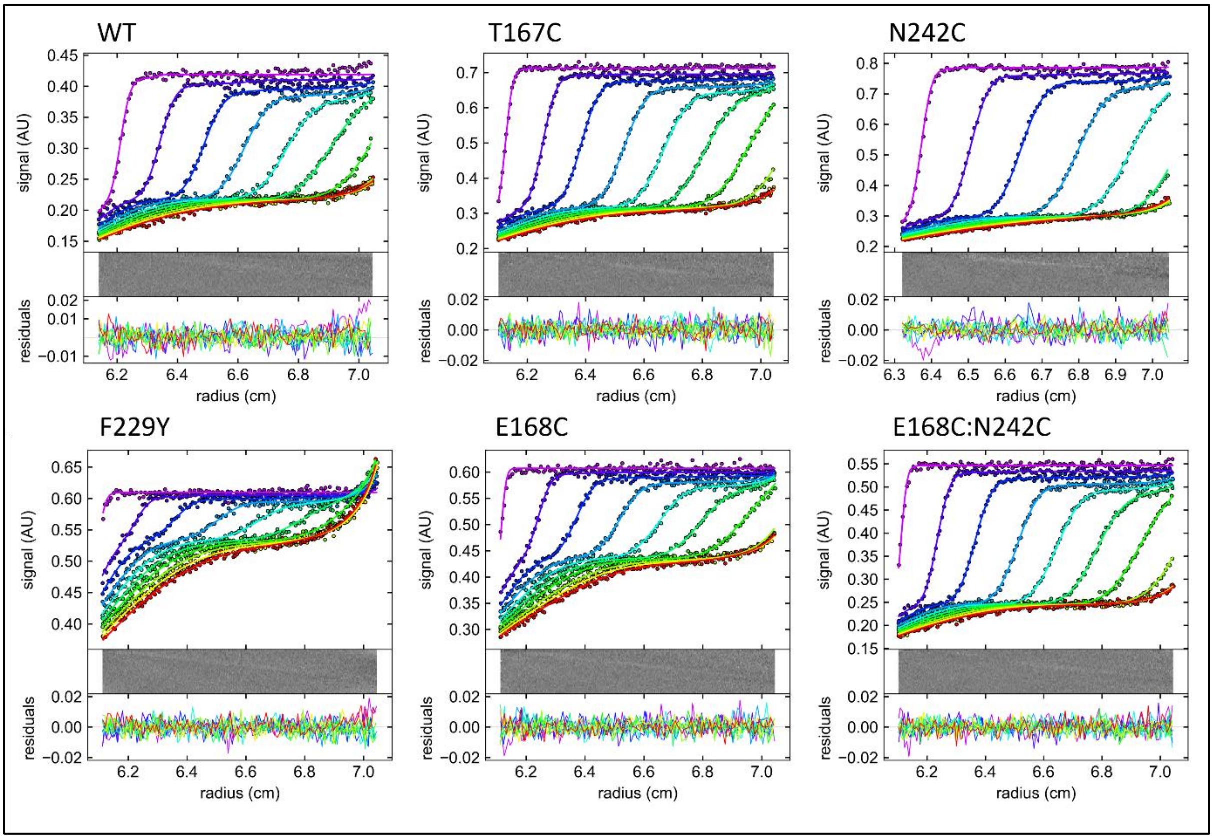

Figure S3. Boundary migration for Vip3Aa activated toxin. Raw representative sedimentation velocity data corresponding to the samples indicated. Each boundary is fitted to a c(s) model, and the residuals are shown in the lower panels.
